# Topographic Factor Analysis: A Bayesian Model for Inferring Brain Networks from Neural Data

**DOI:** 10.1371/journal.pone.0094914

**Published:** 2014-05-07

**Authors:** Jeremy R. Manning, Rajesh Ranganath, Kenneth A. Norman, David M. Blei

**Affiliations:** 1 Princeton Neuroscience Institute, Princeton University, Princeton, New Jersey, United States of America; 2 Department of Computer Science, Princeton University, Princeton, New Jersey, United States of America; 3 Department of Psychology, Princeton University, Princeton, New Jersey, United States of America; University of Maryland, College Park, United States of America

## Abstract

The neural patterns recorded during a neuroscientific experiment reflect complex interactions between many brain regions, each comprising millions of neurons. However, the measurements themselves are typically abstracted from that underlying structure. For example, functional magnetic resonance imaging (fMRI) datasets comprise a time series of three-dimensional images, where each voxel in an image (roughly) reflects the activity of the brain structure(s)–located at the corresponding point in space–at the time the image was collected. FMRI data often exhibit strong spatial correlations, whereby nearby voxels behave similarly over time as the underlying brain structure modulates its activity. Here we develop topographic factor analysis (TFA), a technique that exploits spatial correlations in fMRI data to recover the underlying structure that the images reflect. Specifically, TFA casts each brain image as a weighted sum of spatial functions. The parameters of those spatial functions, which may be learned by applying TFA to an fMRI dataset, reveal the locations and sizes of the brain structures activated while the data were collected, as well as the interactions between those structures.

## Introduction

**Figure 1:**
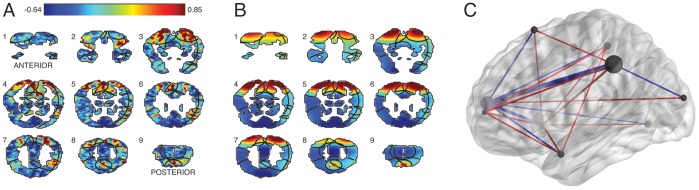
Inferring the hidden structure underlying a set of brain images. **A.** Sample image. A set of coronal slices from a single participant. As indicated by the color bar, high activations (in standard deviation units) are shown in red and low activations are shown in blue. **B.** After applying TFA to the full fMRI dataset, we uncover a set of sources, outlined in black. The coloring in this panel reflects the source weights that best explain the example image shown in Panel A (the same color scale is used in both panels). The sources are also outlined in Panel A, to facilitate comparison. **C.** TFA also reveals interactions between the sources. In this example brain network, inferred from the same participant’s data, each source is represented as a dark gray sphere (whose radius reflects the source’s width), and interactions between the sources are represented by lines. The line thicknesses reflect the strengths of the interactions, where excitatory (positive) connections are shown in red and inhibitory (negative) connections are shown in blue. (To facilitate viewing, we have removed the weakest 60% of the source interactions.) This panel was created using BrainNet viewer [24]. Movie S1 displays a rotating view of the network.

Functional Magnetic Resonance Imaging (fMRI) has revolutionized the field of cognitive neuroscience by allowing researchers to take high resolution three-dimensional snapshots of a person’s brain activity approximately once per second throughout an experiment (Figure 1A). Each *voxel* (the three-dimensional analog of a pixel in a digital photograph) in a collected brain image reflects, roughly, the degree to which the corresponding location in the person’s brain was activated at the time the image was acquired. Each fMRI image comprises tens of thousands of voxels, and hundreds of images may be collected over the course of a single experimental testing session. Researchers rely on these images to gain insights into the brain structures activated during an experiment, the computations those brain structures carry out, and the interactions between the brain structures.

Here we present Topographic Factor Analysis (TFA), a technique for automatically discovering the brain regions that vary their activation during an experiment (Figure 1B) and inferring the network of interactions between those regions (Figure 1C). TFA casts each brain image as a weighted sum of *spatial functions*–parameterized mathematical functions that may be evaluated at arbitrary points in space. The set of spatial functions, which we call the set of *latent sources*, is fixed for a given dataset. The model can then explain each image by activating each source to the appropriate degree. This idea was originally proposed by [1].

The inference problem takes a set of brain images as input and uncovers the most probable source parameters (i.e., their locations and sizes), source weights (i.e., how each image exhibits the sources), and the interactions between sources. In this way, TFA discovers the hidden structure underlying a set of brain images. We have designed our algorithms to scale to large data sets both in terms of the number of images and the number of voxels.

The next section provides a formal description of the modeling assumptions behind TFA. We then describe an efficient algorithm for applying TFA to large fMRI datasets. As a proof of concept, we use TFA to uncover the brain networks underlying a publicly available fMRI dataset collected by [2]. We also discuss the relationship between TFA and closely related approaches including Topographic Latent Source Analysis (TLSA; [1]), Principal Component Analysis (PCA; [3]) and Independent Component Analysis (ICA [4], [5]). We note that TFA can be cast as a special case of TLSA whereby each brain image is treated as independent. We discuss how the efficient algorithm we use to fit TFA to large fMRI datasets may be applied to TLSA via a straightforward modification. Finally, we discuss how TFA may be incorporated into models that seek to leverage both neural and behavioral data to gain insights into cognition.

## Topographic Factor Analysis (TFA)

**Figure 2:**
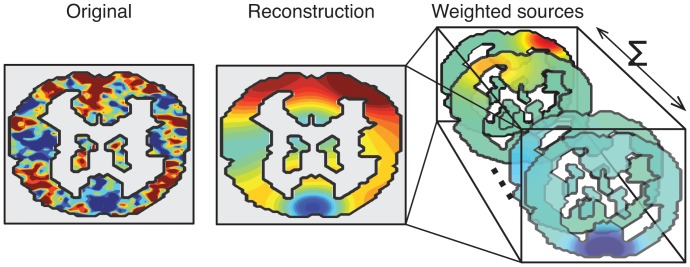
Decomposing a brain image into a weighted combination of sources. A coronal slice from an example brain image is shown on the left. TFA approximates the image as a weighted sum of source images. The approximation (reconstruction) is shown in the middle panel, and several of the (weighted) source images are shown on the right. The color scale is the same as for Figure 1.

TFA assumes that fMRI images reflect the activities of a finite number of sources distributed throughout the brain (Figure 2). (This is a simplifying assumption, of course, but is useful for uncovering hidden structures in brain activity data.) Intuitively, a source could reflect a particular brain structure, or a set of nearby brain structures behaving similarly or carrying out similar computations during an experiment. Each source in TFA is formally defined by a set of parameters of a spatial function. In principle, we may choose any family of spatial functions that describes the sources’ shapes (see *Discussion*). To simplify the presentation, sources in our implementation will be specified as sets of parameters of Gaussian radial basis functions (RBFs). If an RBF has center 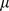 and (log) width 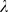, then its activation 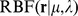 at location 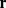 is given by:
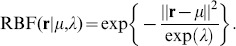
(1)


Thus, each source may be specified using a center parameter 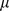 and width parameter 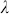. When defined in this way, the sources TFA finds will look “spherical” meaning that they decrease their activation with increasing distance from the source’s center (Figure 2). A source’s width roughly corresponds to a sphere’s radius, in the sense that the width parameter determines how gradually activation falls off with distance from the source’s center. This parameterization allows us to easily interpret each source as a structure or set of structures located roughly at the source’s center, with size roughly proportional to the source's width. The images are then represented as a noisy weighted combination of the sources.

Given these assumptions and a set of brain images, our objective is to compute a conditional distribution over the sources (which are shared across the set) and per-image source weights. This *posterior distribution* will place its mass on the sources and weights that best explain the data. For example, Figure 1B shows the source locations, widths, and weights assigned the highest posterior probability for the brain images in Figure 1A. In general, we use the posterior to calculate interesting patterns in the data, such as the locations and sizes of the structures that vary their activation patterns during an experiment, and the interactions between those structures. The remainder of this section provides a formal definition of TFA and gives our efficient algorithm for estimating the posterior distribution from large datasets of brain images.

### 1.1 The TFA Model

Let 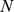 be the number of observed brain images, 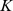 be the number of sources whose parameters we wish to infer, and 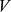 be the number of voxels in each 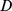-dimensional brain image (for standard fMRI images, 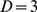). TFA comprises the variables summarized in Table 1 (all are real-valued scalars, unless otherwise specified). Note that the only observed variables are the voxel activations 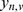. All the other variables are latent variables, whose conditional distributions are to be estimated from the data.

**Table 1:** Variables in TFA.

Variable	Description
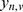	Voxel 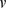 ’s activation in the 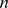 *^th^* image. We use 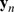 to refer to the 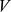 -dimensional vector of voxel activations in image 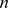 . Let 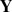 denote the full set of images, 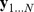 .
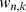	The activation of the 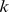 *^th^* source in image 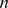 . We use 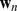 to refer to the 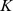 -dimensional vector of source activations in image 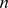 . Let 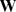 denote the full set of source activation (weight) vectors, 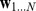 .
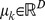	The center of the 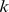 *^th^* source ( 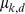 is the coordinate in the 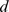 *^th^* dimension). Let 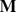 denote the full set of source centers, 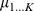 .
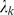	The width of the 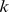 *^th^* source. Let 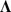 denote the full set of source widths, 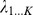 .
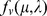	The basis image, specified by center 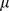 and width 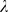 , evaluated at the location of voxel 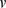 . We use 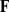 to refer to the 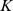 by 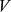 matrix of (unweighted) basis images, specified by 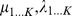 , where the 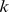 *^th^* row corresponds to the basis images for the 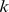 *^th^* source.

TFA defines a joint distribution over the data and latent (unobserved) variables 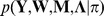, where 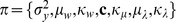 is a set of fixed *hyperparameters* that specify a prior over the distribution of the latent variables (Table 2). To simplify the notation, we suppress the dependence on the hyperparameters from here on.

**Table 2:** Hyperparameters.

Parameter	Description	Value
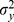	Voxel noise parameter	0.1
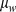	Mean of source weight distribution	0
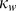	Log precision of source weight distribution	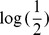
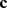	Mean of distribution over source centers	Center of brain image; computed from dataset
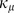	Diagonal of log precision of source center distribution	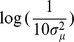 , where 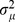 contains the variances across voxel coordinates along each dimension
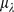	Mean of distribution over source widths	1
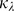	Log precision of distribution over source widths	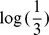

**Figure 3:**
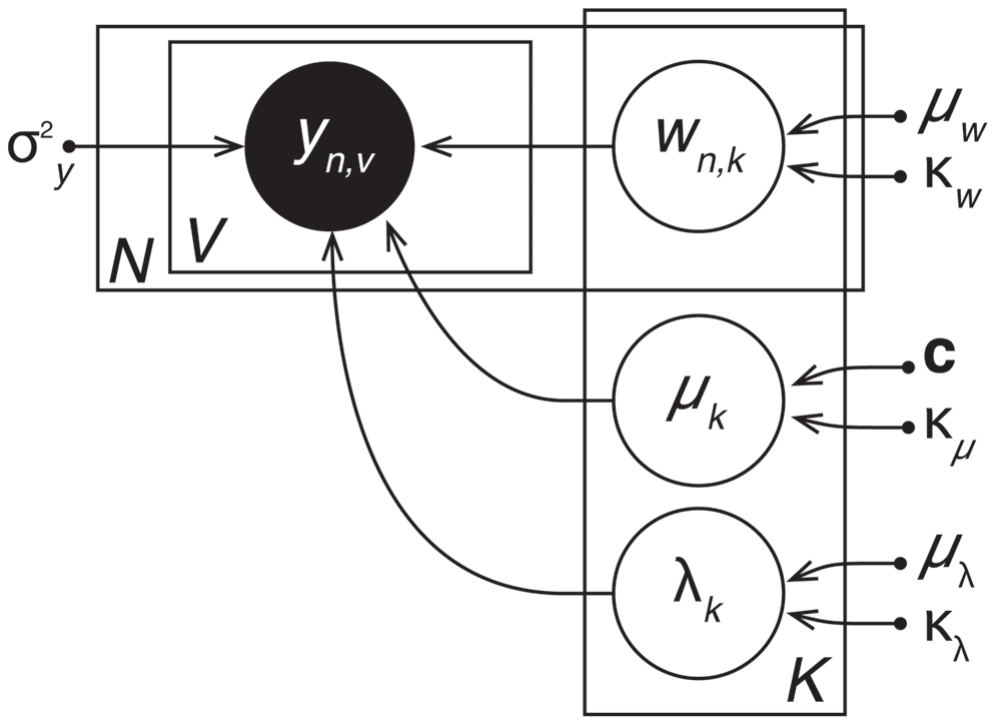
Topographic Factor Analysis. A pattern of 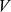 voxel activations is observed during each of 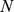 trials (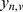). Each of 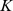 shared sources are defined by their centers (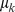) and widths (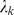). The voxel activations arise due to the sources being activated to varying amounts during each trial, as specified by 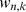. Shaded nodes indicate observed variables, unshaded nodes indicate hidden variables, and dots indicate hyperparameters.

To specify the joint distribution, Figure 3 displays the *graphical model* for TFA. This graph depicts how the joint distribution factorizes into a product of conditional distributions,
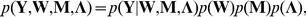
(2)where each node (circle) in the figure represents a variable. Unshaded nodes are hidden variables: 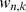 (the weight of the 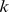 *^th^* source in the 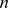 *^th^* image), 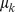 (the center of the 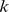 *^th^* source), and 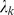 (the width of the 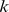 *^th^* source). Shaded nodes are observed variables (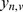 is the activation of voxel 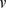 in image 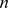). Dots denote the fixed hyperparameters. Arrows denote conditional dependence, originating at terms that appear on the right sides of conditionals and pointing towards terms that appear on the left sides. Rectangular plates denote repeated structure, where the number of copies is indicated within each plate (e.g., 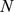, 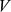, or 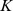). For a comprehensive introduction to graphical models see [6].

We complete the specification of the joint distribution by identifying each factor. The data-generating distribution is
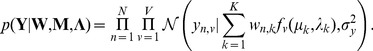
(3)


The distributions of the source weights, centers, and widths respectively are
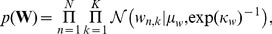
(4)




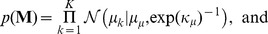
(5)




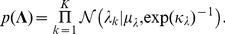
(6)


The factorization of the TFA joint distribution also determines its corresponding *generative process*, i.e., the probabilistic process that TFA assumes generated the data. This process is described in Algorithm 1 (Table 3) which, if implemented, would produce brain images from a TFA model. Specifically, each run generates a single sample from TFA’s joint distribution, yielding one value for each hidden variable and a set of 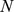 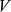-voxel brain images. One perspective on the conditional distribution of hidden variables given the data is that it “reverses” the generative process, finding the distribution of hidden structure that likely produced the observed data. For example, the generative process posits that source locations are drawn from a Multivariate Gaussian (prior) distribution centered on the brain. The goal of posterior inference is to determine which specific sources were most likely sampled from this prior, given the observed brain images.

**Table 3:** Algorithm 1: TFA’s generative process.

** for** *k* = 1 **to** *K* **do**	
	Pick source location 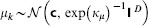 , where **c** is the center of the brain;
	Pick source width 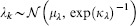 ;
** end**	
** for** *n* = 1 **to** *N* **do**	
	Pick source weights 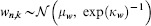 ;
	Pick voxel activation 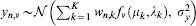 ;
** end**	

Note that we parameterize the variances of the Gaussian distributions using log precision parameters (equal to the log of the inverse of the variance). The log precision parameterization is equivalent to the more commonly used variance parameterization, and facilitates our approximate inference algorithm (Section 1.5).

### 1.2 Computation with TFA

Given data, the main computational goal for TFA is to estimate the posterior distribution of the hidden variables, 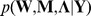.

In theory we could compute this posterior using Bayes’ rule (e.g., [7]):
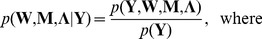
(7)




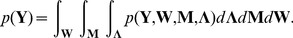
(8)


However, as for many interesting models, computing 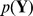 is intractable because it requires integrating over all possible combinations of values that the hidden variables could take on. (This is both analytically difficult and computationally intractable.) Thus, we must develop a method to approximate the posterior. Here we develop a method based on a general approach called *variational inference* [8].

The idea is that we will define a second probability distribution, 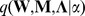, over the hidden variables in TFA. The set of parameters 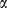 are called *variational parameters* (Table 4), which govern each factor of 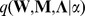, as described in Equations 9 and 10 (below):
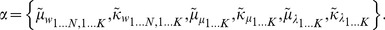
(9)


**Table 4:** Variational parameters.

Parameter	Description
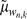	Mean of distribution over source 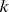 ’s weight in image 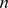
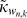	Log precision of distribution over source 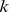 ’s weight in image 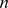
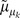	Mean of distribution over source 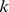 ’s center
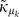	Diagonal of log precision of distribution over source 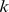 ’s center
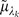	Mean of distribution over source 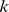 ’s width
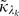	Log precision of distribution over source 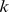 ’s width

We will construct 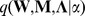 to factorize in a way that allows for straightforward computations. Specifically, we will treat every variable in 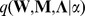 as independent (this is called the *mean field* assumption; [8]):
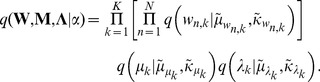
(10)


In our implementation, 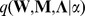 is a product of (independent) Gaussians, where each hidden variable in the model is associated with one of those Gaussians. We tune the parameters in 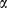 [which govern the means and (co)variances of each factor of 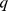] to find a local minimum of the Kullback-Leibler (KL) divergence between 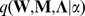 and the posterior distribution over the hidden variables given the data, 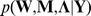:
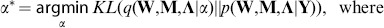
(11)




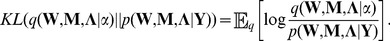
(12)


We noted above that computing the posterior directly (Equation 8) is intractable, and so it seems counterintuitive that we should be able to nonetheless compute the KL divergence between 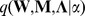 and 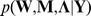. The trick is to instead perform the optimization with respect to 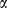 on the Evidence Lower BOund (ELBO), which is equal to the negative KL divergence up to an additive constant [9]:
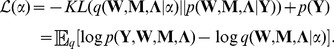
(13)


This casts the posterior inference problem as an optimization problem. Because the ELBO and KL divergence are inversely proportional, a local maximum in the ELBO corresponds to a local minimum in the KL divergence [9]. We proceed by specifying the hyperparameters (prior), initializing the variational parameters (described below), and then iteratively adjusting each parameter in turn. When this inference procedure has converged, we can use the variational distribution 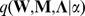 as an approximation of the posterior distribution 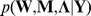.

### 1.3 Setting the Prior

The set of hyperparameters, 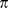, may be adjusted to reflect the properties of the data. We have found the hyperparameter values summarized in Table 2 to work well for several fMRI datasets we examined. To allow the model sufficient flexibility to fit the data, we suggest keeping the prior distribution broad (i.e., by setting the log precision parameters to take on small values, as we have done). When we run the posterior inference procedure, we hold the hyperparameters fixed and update the variational parameters.

The hidden variables in TFA govern the 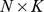 per-image source weights (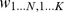) and the 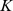 source centers (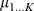) and widths (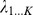). Each of these variables corresponds to a factor of the variational distribution 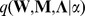, and each factor of 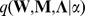 is parameterized by a set of mean and log precision parameters, contained in 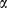 (Equation 10). Each factor of 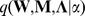 is Gaussian, so the mean of each factor reflects the mean (and expected value) of the corresponding variable, and the log precision of each factor reflects the uncertainty about that variable.

Whereas Gaussian distributions are typically parameterized via mean and variance parameters, we chose to parameterize the Gaussian distributions in TFA using mean and log precision parameters. The intuition driving this design decision is that log precisions have support over the reals, whereas variances have support only over the positive reals. Each update pushes the parameters towards a local optimum. However, because our inference procedure (Section 1.5) is based on stochastic optimization [10], any given update may not increase the objective. Utilizing parameters that do not have range restrictions avoids parameter drift into undefined regions of parameter space [11]. (This is also why we parameterize each source’s RBF with its log width, rather than specifying the width directly.).

### 1.4 Initializing the Variational Parameters

We initialize the log precisions of each factor of 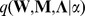 as follows:

- Log precisions of distributions over source weights: 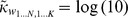.
- Log precisions of distributions over source centers: 
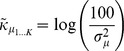
 (see Table 2).
- Log precisions of distributions over source widths: 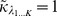.

The variational objective in Equation 13 has many local optima, and practical applications of variational inference must address this issue. Typically this is done in one of two ways. The simplest is to run several random restarts of the algorithm from randomized initial parameters. A more complex, but often more effective, approach is to design domain-specific techniques to initialize the variational parameters that start the algorithm in a place that tends to lead it to good local optima. For TFA, we explored both approaches.

To randomly initialize the means of each factor of 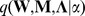, we simply draw these parameters from their associated prior distributions by running the generative process (Algorithm 1, Table 3) a single time. We describe a domain-specific initialization technique, termed *hotspot initialization* in the next sub-section.

#### 1.4.1 Hotspot initialization

**Figure 4:**
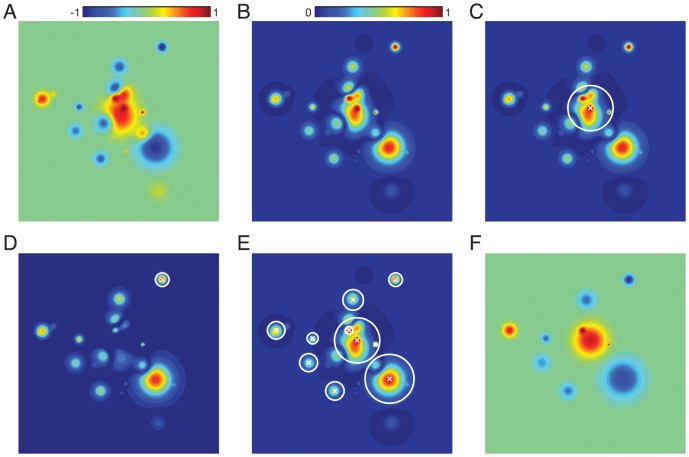
Initializing source centers, widths, and weights. Here we illustrate the initialization procedure for a synthetic 2-dimensional example image. **A. The original mean image.** The mean is taken across observations in the dataset. **B. The centered and folded mean image.** This image was generated by subtracting the mean and taking the absolute value of the image in Panel A. Note that Panels B - E use a different color scale than Panel A and F, as shown by the color bars. **C. Fitting the first source.** We begin by placing the first source’s center at the location at which the folded image displays maximal activation. We then adjust the source’s width using convex optimization. The white level curve, which indicates the locations at which the source’s value is 0.1, is used to illustrate the fitted source’s width. **D. Fitting subsequent sources.** Subsequent sources are fit using the same procedure, but on the residual image (after previous sources have been subtracted off). Here, the source localized and sized using the original image has been subtracted off, leaving a “hole” in the image. The next hotspot appears at a different location, as shown by the newly placed white level curve. **E. The full procedure.** The process of iteratively fitting sources to the residual images continues until 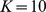 sources are placed. Note that the original synthetic image was constructed using 25 sources, and thus some regions of the image are not explained by the fitted sources. **F. The reconstructed image.** The source weights are estimated using linear regression. This panel uses the same color scale as Panel A.

Hotspot initialization places and sizes sources using the areas of very high and low activation, termed *hotspots*, in the mean image (where the mean is taken across observations in the dataset). We illustrate how this process works in Figure 4. After computing the mean image (Figure 4A), we center it by subtracting the mean activation, and then “fold” the image by taking the absolute values of all of the activations. The result is a set of non-negative activations, where both the highest and lowest activations in the original mean image appear as peaks in the centered and folded image (Figure 4B). Using this folded image, we place the mean of each source center distribution (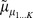), one at a time, at the locations of these peaks (i.e., hotspots). After placing each source’s center, we adjust the mean of its width distribution (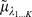) using convex optimization (Figure 4C). Specifically, we find
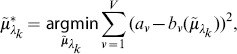
(14)where 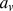 is the activation of voxel 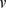 in the folded image, and 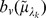 is the activation of voxel 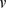 in the source image (i.e., RBF) constructed using mean 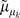 and width 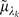. We next create a source image by evaluating the activation of the source (given the center and width parameters) at the location of each voxel in the brain image. We subtract the source image from the brain image; the resulting residual image contains the brain activations that are left unexplained by the source. We then fit the next source’s location and width using the residual image (Figure 4D). This process of fitting sources to the residual brain images continues until 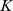 sources (with 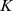 specified in advance) are placed (Figure 4E).

Initializing the 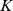 source centers and widths as described above gives us a point estimate of the source image matrix 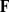. To obtain 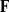, we simply fill in each row, 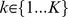, by evaluating a radial basis function (whose parameters are the 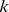 *^th^* source’s center and width) at the location of each voxel. Although this point estimate of 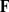 was obtained using only the mean brain image, we can use it to initialize the per-image source weights for *all* of the brain images. To initialize the source weights, we can leverage the fact that TFA casts voxel activations as draws from Gaussian distributions whose means are linear combinations of RBF sources (see Algorithm 1, Table 3). In expectation, the 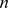 *^th^* vector of voxel activations is given by
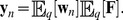
(15)


We know 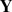 (i.e., the set of 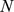 observed brain images), and we can approximate 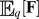 using our point estimate of 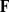. Therefore we can solve for the source weight matrix 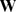:
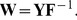
(16)


We then initialize 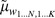 using the corresponding entries of 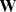. Note that although we obtain the point estimate of 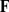 using only the mean image, the per-image weights are initialized using the full set of images.

The hotspot-initialized parameter values often provide a good fit to the original brain images (e.g., compare Figures 4A and 4F). However, because the estimated source centers and widths take only the mean brain image into account (rather than the individual images), important information may be missed by the initialization procedure. We next describe how we tune the variational parameters, 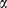, to best explain the full set of observed brain images.

### 1.5 Optimizing the Variational Objective

Our goal is to adjust the variational parameters to maximize the ELBO (Equation 13), thereby minimizing the KL divergence between the variational approximation 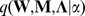 and the posterior distribution 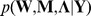. We use the stochastic optimization procedure described by [12] to maximize the ELBO. Specifically, with each update we approximate the ELBO by drawing 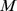 samples, 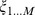, from 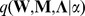:
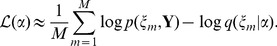
(17)


Note that as 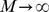 the approximation becomes exact. Each sample from 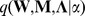 contains 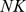 source weights (one weight per source, per image), 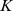 source centers, and 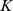 source widths. We use stochastic gradient ascent to find a local maximum of the ELBO by repeatedly sampling from 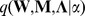, computing the gradient of the ELBO with respect to 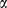, and updating 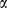 by taking a small step in the direction of the gradient.

The gradient of the ELBO with respect to the 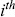 variational parameter, 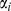, may be estimated as follows [12]:
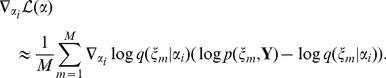
(18)


The gradients of 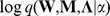 with respect to each element of 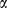 may be found in Materials S1.

As shown by [12], the expectations of these estimates of 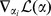 are the true gradients. However, because the estimates are obtained by drawing random samples from 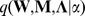, any given estimate will vary from the true gradient. We can use mathematical constructs called *control variates* to reduce the variance of the estimates of each 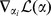 while simultaneously ensuring that the expectations of the estimates are equal to the true gradients (for a more detailed explanation and derivation, see [12]):
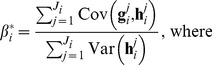
(19)

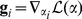
(20)

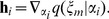
(21)


Here the superscript 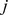
s denote the 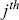 dimension of the corresponding vectors. As outlined in Algorithm 2 (Table 5), we subtract 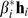 from the estimate of 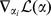 to obtain a new, more reliable, estimate of the gradient.

**Table 5:** Algorithm 2: Variational inference procedure for TFA.

**Input** : A set of *N* images, **Y**; a specified number of sources, *K*; and a set of hyperparameters, *π*					
**Output**: A set of fitted parameters, *α*					
*t* ← 0;					
*maxStepSize* ← 1;					
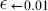 ;					
*α* ← initializeParameters(**Y**, *π*);					
*L* ← length(*α*);					
*η* ← zeros(*L*);					
*M* ← 500;					
**while** *not DONE* **do**					
		*t* ← *t* +1;			
		*α*_0_ ← *α*;			
		*ξ* ← sampleFromQ(*M*, *α*_0_, *π*, *i*);			
		**for** *i* ← 1 **to** *L* **do**			
			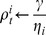 ;		
			**for** *m* ← 1 **to** *M* **do**		
				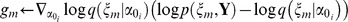 ;	
				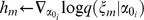 ;	
			**end**		
			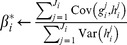 ;		
			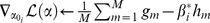 ;		
			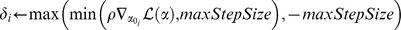 ;		
			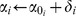 ;		
			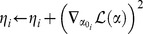 ;		
		**end**			
		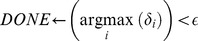 ;			
	**end**				

Finally, for each iteration of the inference procedure (whereby we update all parameters in 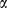), we assign a per-parameter learning rate, 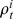, to each parameter in 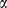 using an adaptive subgradient method [12], [13]. This learning rate changes with each iteration; in the 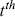 iteration the learning rate for 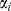 is:
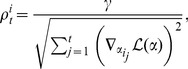
(22)where we set 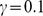 in our implementation, and where 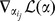 is the estimate of 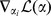 in the 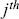 iteration. This learning rate is multiplied by the gradient prior to updating each parameter in the direction of its gradient. In other words, it scales the size of the steps the inference procedure takes.

Our complete inference procedure is outlined in Algorithm 2 (Table 5). Note that each iteration of the outer for loop (over each element of 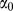) does not depend on the updates performed in the other iterations. Therefore, if one has access to a cluster of compute nodes with shared access to a common filesystem, one may perform these length(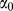) updates in parallel, thereby substantially speeding up the computation.

### 1.6 Subsampling

Each iteration of the inference algorithm (Algorithm 2, Table 5) requires updating each of the 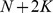 sets of variational parameters contained in 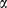. Further, updating each of the 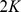 sets of global parameters (governing the source centers and widths) requires performing computations on the full set of 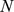 images. For each of these updates, we must also examine the full set of 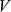 voxels that the images comprise.

Considering the full dataset with each update seems inefficient. For example, suppose that we were to consider only 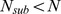 of the images contained in our dataset. We might still be able to improve our estimates of the source centers and widths, even though we had not seen *every* image. Similarly, even if we considered 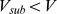 of the voxels in the images (rather than all 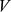 of the voxels), we might nonetheless be able to gain some insights into the hidden variables. We can use a technique called *stochastic variational inference* [14] to leverage these intuitions, thereby substantially reducing the number of calculations we need to perform during each update. Specifically, we can perform *image-level* subsampling and *voxel-level* subsampling as described below. Subsampling allows us to apply our inference procedure to the large datasets prevalent in neuroscientific research using commonly available computing hardware in a reasonable amount of time.

#### 1.6.1 Image-level subsampling

We implement image-level subsampling by selecting a new subset of 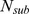 unique images to be considered during each iteration of the while loop in Algorithm 2 (Table 5). Note that the same 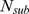 images must be used to update all of the parameters during a given iteration of the while loop. Also note that we will not be able to gain any insights into the local parameters (i.e., the per-image source weights) associated with the 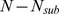 remaining images, so updates will not be performed for those local parameters. We will need to adjust how we update the remaining parameters (via the gradient of the ELBO; Equation 18) to account for the fact that we are not considering all of the images available to us. The affected terms that must be modified include 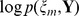, 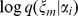, and 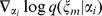 (see Materials S1 for details on computing these terms, which are used in Algorithm 2 in Table 5. We found 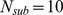 to provide a good balance between speed (which is maximized by reducing 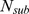) and accuracy (which is maximized by increasing 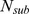).

#### 1.6.2 Voxel-level subsampling

We implement voxel-level subsampling by randomly selecting a new subset of 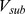 adjacent (contiguous) voxels to be considered during each iteration of the while loop in Algorithm 2 (Table 5). As for image-level subsampling, it is important that the same voxels be used to update all of the parameters during a given iteration of the while loop. Voxel-level subsampling affects only the computation of the 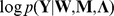 factor of 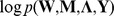, which reflects the likelihood of the observed images given the hidden variables (Equation 3). Because this term sums over 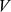 voxels, we must account for the fact that we are considering only 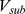 voxels by scaling by 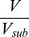. (This ensures that the stochastic gradient remains unbiased.) We found 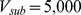 to provide a good balance between speed and accuracy. (The images in the datasets we examined contained on the order of 30,000 voxels.).

### 1.7 The Final Update

During the last iteration of the while loop in Algorithm 2 in Table 5 (i.e., when the ELBO has reached a local optimum), we need to account for the fact that, because we drew a random subset of images with each update, the inference procedure may not have considered every image. If so, the local variables (per-image source weights) associated with the left-out images will not have been updated from their initialized values. To ensure that all of the per-image source weights converge to local optima, we set 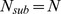 and 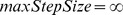, fix all of the global parameters, and re-run the inference procedure until all of the local parameters converge. Note that although we cannot use image-level subsampling to fit the full set of per-image source weights (since each image must be considered in order to determine its associated source weights), image-level subsampling allows the inference procedure to converge more rapidly on the global parameter estimates (i.e., the source centers and widths).

### 1.8 A Useful Approximation for Updating the Source Weights

We found that when we initialized the per-image source weights as described in Section 1.4.1 (i.e., when we solved for the most likely weights using linear regression; Equation 16), the source weight estimates were nearly always initialized to a local optimum. We determined this empirically by first computing the ELBO given the expectations of the initialized parameters (Equation 13). We then took independent draws from 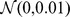, added those draws to each entry of the weight matrix 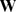, and re-computed the ELBO given those new weights. We repeated this procedure 100 times (each time resetting the weights to their initialized values before adding the random noise) and found that the ELBO decreased in every case, implying that the initialized values reflected a local maximum. (In contrast, the source centers and widths were not typically initialized to local optima, probably because the initialization procedure for the source centers and widths considers only the mean image rather than each individual image.) We leveraged this finding to substantially improve our algorithm’s convergence properties by re-initializing the weights after updating *any* of the source centers or widths. Continually updating the source weights in this way also appeared to reduce the likelihood of the inference procedure getting stuck in poor local optima.

## Results

Our objective in fitting TFA to a set of brain images is to discover the hidden structure underlying those images. In particular, we wish to identify the locations and sizes of sources (which reflect one or more brain structures or substructures), the per-image source weights (which reflect the degree to which each source is activated in each image), and the correlations between source weights across images (which we interpret as reflecting the extent to which the sources interact). In this way, we can use the structure that TFA uncovers to help make sense of complex fMRI datasets.

TFA casts brain images as weighted sums of spatial sources. In Section 1.2 we described how to apply TFA to a dataset by approximating the posterior distribution over the source centers, widths, and weights, given an fMRI dataset. We sought to both evaluate the quality of this posterior and to use the posterior to gain insights into an fMRI dataset.

We applied TFA to an fMRI dataset collected by [2]. The dataset comprises data from 9 participants who each viewed 6 presentations of each of 60 line drawings, for a total of 360 viewings (each with an associated brain image). The drawing presentations were organized into 6 *epochs*, where all 60 drawings were presented in a random order during each epoch. The participants were instructed to think about the meaning of the word associated with each drawing as they viewed it. The drawings were selected from 12 categories: animals, body parts, buildings, building parts, clothing, furniture, insects, kitchen items, man made objects, tools, vegetables, and vehicles.

### 2.1 Visual Inspection of the Reconstructed Images

Figure 1A displays coronal slices from a single brain image, taken from one participant as they viewed the word “refrigerator.” Figure 1B displays a reconstructed version of the same image under TFA’s posterior (using 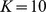 sources). To make the reconstructed image, we computed the source centers, widths, and weights that were assigned the highest posterior probability after applying TFA to the participant’s data. We used these source centers and widths to construct a source image matrix, 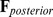, and computed a weighted sum of the rows of 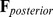 to reconstruct each brain image in the dataset. The black curves overlaid on the brain slices denote the contours of the 10 source images. (Note that not all sources appear in each slice.).

**Figure 5:**
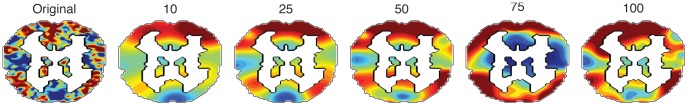
Sample reconstructions using different numbers of sources. A coronal slice from one participant’s brain image is displayed on the left. Moving from left to right, each coronal slice displays the associated TFA reconstruction using the indicated number of sources. The color scale for all panels is the same as for Figure 1.

Comparing the images in the original dataset with their associated reconstructions can tell us about the qualitative aspects of the data that TFA fits well, and also about the aspects of the data that TFA does not fit well. The reconstructed image shown in Figure 1B looks qualitatively similar to the corresponding original image in Figure 1A, indicating that the inference procedure has converged to a reasonable local optimum. Comparing the images visually reveals that the reconstruction has maintained the low spatial frequency information, but not the high frequency information, in the original image. For example, the dorsal (top) edges of slices 3–8 in the original brain image (Figure 1A) display large contiguous patches of high activation (red). These patches also appear in the reconstructed image (Figure 1B). However, whereas these high activation patches are also visible in slices 1 and 2 of the reconstructed image, they are not visible in slices 1 or 2 of the original image. This is because the sources used to explain these patches, being spherical, extend some of their mass into slices 1 and 2, whereas the patches in the original image are irregularly shaped. TFA’s ability to fit high spatial frequency information within a given dataset is constrained by the shapes of the sources and the number of sources we wish to fit (Figure 5).

### 2.2 Explaining and Predicting the Data Covariance Matrix

**Figure 6:**
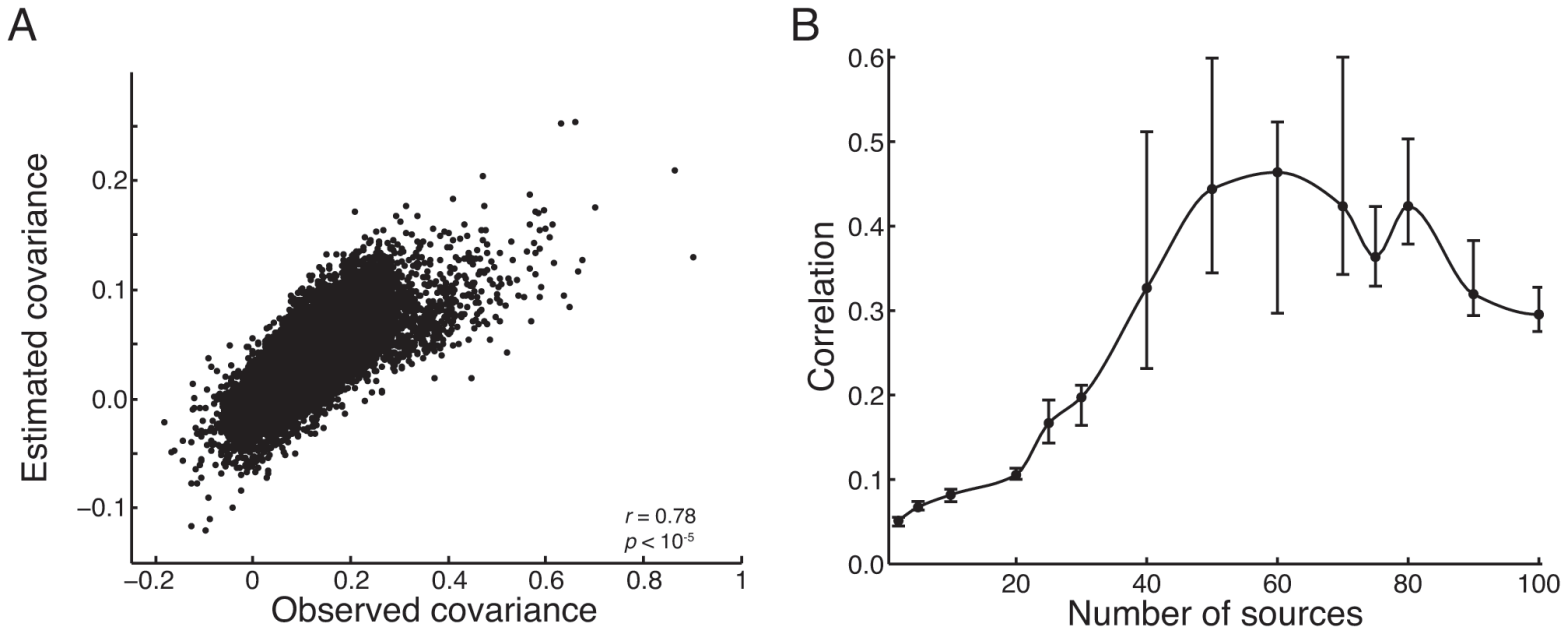
Predicting the covariance structure of an fMRI dataset. **A.** Each dot reflects the covariance between a pair of images from a single participant (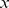-axis: observed, 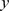-axis: estimated) using 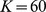 sources. The correlation reported in the panel is between entries in the two covariance matrices. **B.** We also used TFA to estimate the covariance structure of held-out data, using a 6-fold cross validation procedure. The panel displays the median correlations (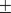 bootstrap-estimated 95% confidence intervals) between the observed and estimated covariance matrices (of held out data), as a function of the number of sources we fit. The medians are taken across the 6 folds and 9 participants, and the error bars reflect across-participant variability.

In addition to examining the quality of individual image reconstructions, we may also wish to know the extent to which TFA preserves the covariance structure across all of a participant’s images. As shown in Figure 6A, we computed the observed across-image covariance matrix for one participant and compared it to the TFA-estimated across-image covariance matrix (using 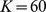 sources). Each dot in the figure reflects a single entry in one of these 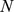 by 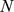 covariance matrices (correlation between entries in the observed and estimated covariance matrices: 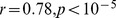).

Because each source in TFA is a spatial function, once we know the parameters for each source (by applying TFA to an fMRI dataset) we can evaluate those functions at any location in space, including locations that were not included in the training set. This is useful, for example, if we wish to correct corrupted voxels in a given image or compare images taken at different sampling resolutions. We used a cross validation procedure to assess the extent to which the predicted activations for held-out voxels preserved the covariance structure of the true activation patterns of those voxels. This procedure provides insights into how well TFA’s reconstructions generalize to new observations. We repeated the procedure for a range of values of 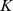 (number of sources), which also tells us about the number of sources we should fit to the dataset.

We ran the cross validation procedure separately for each participant. We began by assigning each image to one of six folds, such that each fold contained exactly one presentation of each word (i.e., 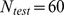 images). For each fold, we estimated 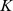 source centers and widths by applying TFA to the out-of-fold images. Next, we randomly assigned voxels to each of two equally sized groups. We fit the source weights using the in-group voxels from the in-fold images. We computed the expected activations of the out-of-group voxels in the in-fold images, and computed the across-image covariance matrix of those estimated activations. We then compared the observed and estimated across-image zcovariance matrices (for the out-of-group voxels in the in-fold images). We repeated this procedure 12 times (once for each image fold and voxel group) to obtain a distribution of correlation coefficients for each value of 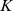, for each participant.

As shown in Figure 6B, TFA achieved a peak (median) predictive correlation of 0.45, using 60 sources. This indicates that fewer than 60 sources do not sufficiently capture the complex underlying spatial structure of the brain images. When we used more than 60 sources, the model failed to generalize as well to new data, suggesting that TFA was overfitting the training data. In this way, cross validation may be used to determine the ideal number of sources for a given dataset. Further, the analysis shows that the image representations of held-out data, estimated using TFA, accurately reflect the covariance structure of the original images (i.e., the correlations are substantially greater than 0 as the numbers of sources used to fit the model vary over a wide range).

### 2.3 Category-specific Brain Networks

The above analyses indicate that TFA yields good fits to fMRI images (e.g., compared via visual inspection) and reliably estimates the covariance structure of held out data. We next sought to use TFA as an exploratory tool for finding interesting patterns in the fMRI data.

FMRI investigations have traditionally searched for univariate [15]–[17] and multivariate [18] differences in brain activations across conditions in an experiment. Over the past several years, neuroscientists have also become increasingly interested in measuring functional connections between brain structures [19], [20]. So called *network connectivity analyses* typically use the voxel-by-voxel covariance matrix (taken across images) to infer the strengths of connections between those voxels. However, computing such matrices entails a substantial computational burden. For example, the covariance matrix of a 50,000 voxel brain image contains 2.5 million entries, and occupies nearly 20 GB of memory (using double precision). Manipulating many such covariance matrices or performing *post hoc* analyses, such as regressions, that require 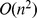 memory can become unwieldy on modern hardware. Consequently, researchers interested in brain connectivity often focus on a set of preselected regions of interest.

TFA provides an alternative means of examining brain networks that does not require preselecting regions of interest. Applying TFA to a dataset yields a set of 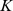 sources (with 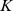 selected in advance by the practitioner, e.g., using cross validation as in Figure 6), each corresponding to a specific brain region or set of regions. Importantly, these sources are determined solely from the data and may be located anywhere in (or around) the brain. [Note that sources need not be located solely in grey matter– they may be located in white matter, cerebrospinal fluid, or outside of the brain. For example, a patch of brain activation near the cortical surface might be well explained by a source placed in the center of that patch (e.g., in gray matter), or it could be well explained by a sufficiently wide source placed outside of the brain, but near the patch. One should therefore interpret a source as reflecting the activities of brain structures over which it spreads its mass rather than as a single point.].

The across-image covariance of the weight matrix 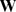 specifies how similarly or differently each source behaves from image to image. We can use the covariance of 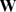 to estimate the signs and strengths of the interactions between each pair of sources, just as standard connectivity analyses use image covariance matrices to estimate interactions between pairs of voxels [20]. In this way, the covariance of the weight matrix provides a compact representation of the full brain connectivity matrix that may be easily interpreted, viewed, and manipulated.

**Figure 7:**
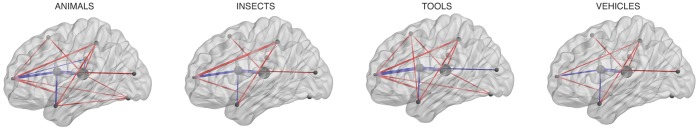
Brain networks underlying category representations. Each panel reflects the inferred brain network from one participant’s data as they viewed words from the indicated category. (Connections with absolute strengths less than the 80^*th*^ percentile strength from the animals network are omitted for visualization purposes; no thresholding was used in our statistical tests of network reliability.) Movies S2–S5 display rotating views of these networks.

As a proof of concept, we provide one example of an exploratory analysis that may be performed using these inferred brain networks in Figure 7. Each panel of the figure reflects the inferred brain network from one participant’s data as they viewed words from the indicated categories. We show networks derived using 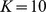 sources in the figure to facilitate visualization, but for the analyses that follow we used 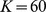 sources (chosen using the cross validation procedure described above and depicted in Figure 6B). After applying TFA to the participant’s data, we computed the covariance of the source weight matrix across presentations of words within each category to infer the source interactions.

We performed a split-half analysis to assess the reliability of the category-specific networks we inferred, as follows. After applying TFA with 60 sources to each participant’s data, we divided the data into *odd* epochs (i.e., the first, third, and fifth set of presentations of the 60 drawings) and *even* epochs (i.e., the second, fourth, and sixth set of presentations). We then computed the covariance of the corresponding epochs’ rows of 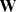 to infer each participant’s category-specific networks, for both the odd and even epochs. This yielded two inferred networks per category.

We computed a confusion matrix containing, for each pair of categories, the correlations between the off-diagonal entries of each category’s covariance matrix (which reflects the interactions between the sources) from the odd epochs and the off-diagonal entries of the covariance matrices from the even epochs. The diagonal entries of the confusion matrix reflected correlations across runs between networks of the same category, and the off-diagonal entries reflected correlations between networks of different categories. We used a permutation test to ask whether the correlations along the diagonal were reliably stronger than the off-diagonal correlations. We first took the mean confusion matrix across participants, and used a 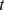-test to compare its on- and off-diagonal entries. We then estimated a null distribution of 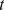-values by repeating the analysis 1,000 times, shuffling the rows of the confusion matrix each time [21]. The observed across-participant 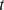-value was larger than 99.33% of the shuffled 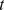-values (i.e., 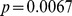), indicating that the networks we inferred for the same category were reliably more similar (across runs) than the networks we inferred for different categories.

## Discussion

TFA reveals the locations and sizes of sources of brain activity that underly an fMRI dataset, as well as the interactions between those sources. TFA identifies these sources by decomposing fMRI data into weighted sums of spatial functions. Applying TFA to a dataset yields a conditional distribution over parameters (i.e., centers and widths) of each source, and the per-image source weights given the brain images. The covariance of the source weights across images provides information about the interactions between the sources.

We demonstrated that reconstructed images, created by computing weighted sums of the sources’ images (i.e., the activations of the sources at the location of each voxel), preserve the low spatial frequency content of the original images. TFA also preserves the covariance structure of a dataset across images and can accurately predict the covariance structure of held-out voxels. Finally, we demonstrated how TFA may be used to discover networks of sources that reflect thoughts about a specific stimulus category.

### 3.1 Relation to Other Techniques

**Figure 8:**
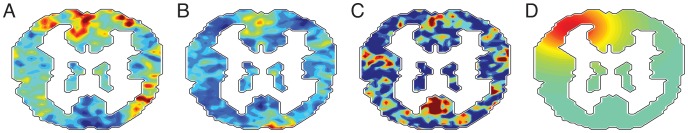
Factors. **A.** Sample image. One coronal slice of a single brain image; high activations are shown in red and low activations are shown in blue. Examples of factors obtained using (**B**) PCA, (**C**) ICA, and (**D**) TFA are shown in the panels. The color scale for all panels is the same as for Figure 1.

By decomposing fMRI data into weighted combinations of spatial functions (Figure 2), TFA reveals some aspects of the structure underlying a dataset. Standard techniques, such as Principal Component Analysis (PCA; [3]) and Independent Component Analysis (ICA; [4], [5]) are closely related to TFA. For example, PCA may be used to obtain a set of factors that best explain the covariance structure of a set of observations, and ICA may be used to determine a set of distinct features that underly those observations (Figures 8B, C). Each observation in the original dataset may then be approximated by a weighted sum of a subset of those factors.

Factors obtained using PCA and ICA are themselves images of the same size as the images in the original dataset (i.e., each PCA and ICA factor is a 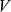-dimensional vector). In contrast, TFA factors are constrained to have a specified functional form; in our implementation, each factor was defined by a set of radial basis function parameters. Constraining TFA’s factors to have a given functional form substantially reduces the freedom TFA has to explain the dataset, which in turn reduces the fidelity of the representations. However, this reduction in reconstruction performance buys interpretability: whereas PCA and ICA factors are not directly interpretable, each TFA factor is easily interpreted through its set of parameters (e.g., its center and width parameters). In addition, TFA may be used to predict the activations of held-out voxels using their locations (e.g., Figure 6), whereas PCA and ICA cannot.

TFA is also closely related to Topographic Latent Source Analysis (TLSA; [1]). Like TFA, TLSA also defines each factor via a set of parameters to a spatial function, and therefore TFA and TLSA both benefit from having interpretable and resolution-independent factors. From a matrix factorization perspective, TFA decomposes a matrix of brain images 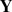 into the product of a source weight matrix 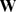 and a source image matrix 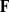:
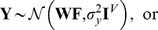





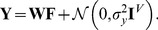
(23)


TLSA performs an additional decomposition on 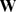:
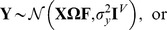





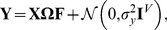
(24)where 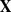 is called the *experimental design matrix* and 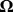 is the *covariate-source loading matrix*. The 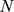 by 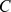 experimental design matrix describes the extent to which each of 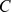 covariates of interest were activated as each image was collected. For example, a given row of 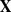 might correspond to an indicator vector denoting which of several experimental conditions the image is associated with, or a vector representation of the word the participant was viewing while the image was collected. Each column of 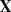 reflects a different covariate, such as a particular category or semantic feature. The (unobserved) 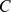 by 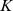 covariate-source loading matrix 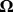 describes how each of the 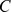 covariates affects the activations of each of the 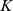 sources (where sources in TLSA are defined as in TFA). In this way, TLSA builds on the intuition that images collected during similar experimental conditions (e.g., while the participant was thinking about the same word) will be similar. The sources that TLSA finds are biased towards brain regions that exhibit the same covariance structure as 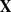.

When 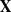 is equal to the 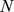 by 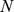 identity matrix, 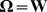. In this way, TFA may be considered as a special case of TLSA, where 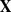 is the identity matrix (i.e., each image is treated as independent). By treating images as independent, TFA is able to uncover interactions between sources (via the covariance of 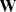). Defining 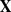 to be anything other than the identity matrix (as in the general formalization of TLSA) forces the source interactions to precisely mirror 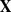, precluding the identification of interesting interactions between sources.

### 3.2 What Other Types of Data could TFA be Applied to?

In principle, TFA may be applied to any spatial dataset–that is, any dataset whose observations comprise sets of value-location pairs (e.g., brain images, photographs, video, geolocation data, motion tracking data, etc.). However, in practice TFA will likely provide useful information only when the data conform to the general assumptions underlying TFA’s generative process (Algorithm 1 in Table 3). Specifically, TFA assumes that sources are shared across observations and contribute to each observation to a varying degree. Brain data satisfy this assumption especially well: intuitively, each source reflects the activity of a set of nearby brain structures or substructures, which remain at the same locations within the participant’s brain over the course of the experiment. Examples of spatial data that we expect TFA to perform well on include:

- Neural recordings [e.g., (functional) Magnetic Resonance Imaging, intracranial and scalp electroencephalography, magnetoencephalography, etc.]. The sources will reflect brain structures and substructures that vary their activation during the recordings.
- Photographs taken from a fixed location. Sources will reflect structures that are common from image to image.
- Sensory measurements (e.g., seismic data). Sources will reflect activity hubs (e.g., hubs of seismic change).

We do not expect TFA to perform well on datasets where the underlying structure is not held constant (or not measured) across observations. Examples include:

- Geolocation data (e.g., cloud movement, GPS tracking, etc.).
- Sets of photographs taken from different locations.
- Tracking systems (e.g., radar).

### 3.3 Extending TFA

TFA makes a number of simplifying assumptions that may be worth examining further in future work. For example, in our implementation, each source is an RBF, and each participant and image is treated independently. Here we propose several ways in which these simplifications may be relaxed.

#### 3.3.1 Source shapes

As illustrated in Figure 1, contiguous patches of activation in fMRI images can be irregularly shaped. As more sources are added, TFA explains high spatial frequency information in the images with increasing accuracy (Figure 5). (In the limit, where each voxel has its own source, this is mechanically true.) However, we might benefit in computational efficiency from allowing sources to take more complex shapes.

For example, rather than specifying one width parameter per source (i.e., forcing sources to be spherical), one could specify, for each source, one width parameter for each dimension (resulting in ellipsoid sources in 3-dimensional images). This would allow sources to expand or contract along each dimension to better explain patches of brain activity. One could also implement multivariate Gaussian sources, which would result in sources that would appear as oriented ellipsoids. Further, one could model each source as a weighted combination of Gaussians by fitting the parameters of 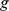 Gaussians (for each source), and also fitting a set of 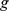 mixing parameters (for each source) describing the relative activations of each source’s components. As 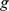 increased, each source’s shape would become more complex, allowing TFA to fit more complex patterns (i.e., patterns at higher spatial resolutions) with fewer sources. However, these benefits do not come for free: as the source shapes become more complex, each source becomes more difficult to interpret and more parameters must be estimated.

#### 3.3.2 Hierarchical extensions

In our implementation, we applied TFA to each participant’s data individually, which was sufficient for demonstrating our approach. However, future work may benefit from a hierarchical implementation of TFA, whereby each participant’s data are treated as perturbations of a global template [22]. This would facilitate comparisons across participants, and would also allow for hypothesis testing on the locations of specific sources and source interactions. A hierarchical approach may also allow our inference procedure to find better local optima, especially in noisy data, to the extent that different participants’ data are similar. This is because ambiguities in one participant’s data may be resolved by examining another’s data.

### 3.4 Integrating TFA into Behavioral Models

TFA defines a probability distribution whose draws are brain images. When we apply TFA to a dataset by performing posterior inference, we uncover the most probable hidden variables that produced the observed data. In other words, we uncover the distribution, within the family of distributions defined by TFA, that takes into account our observed brain images. Sampling from this distribution (using TFA’s generative process; Algorithm 1 in Table 3) yields sets of brain images that look similar to the original dataset (where the degree of similarity depends on the number of sources; Figure 5). This distribution over brain images can be treated as any other probability distribution and thereby integrated into more complex models that seek to incorporate neural data [23].

**Figure 9:**
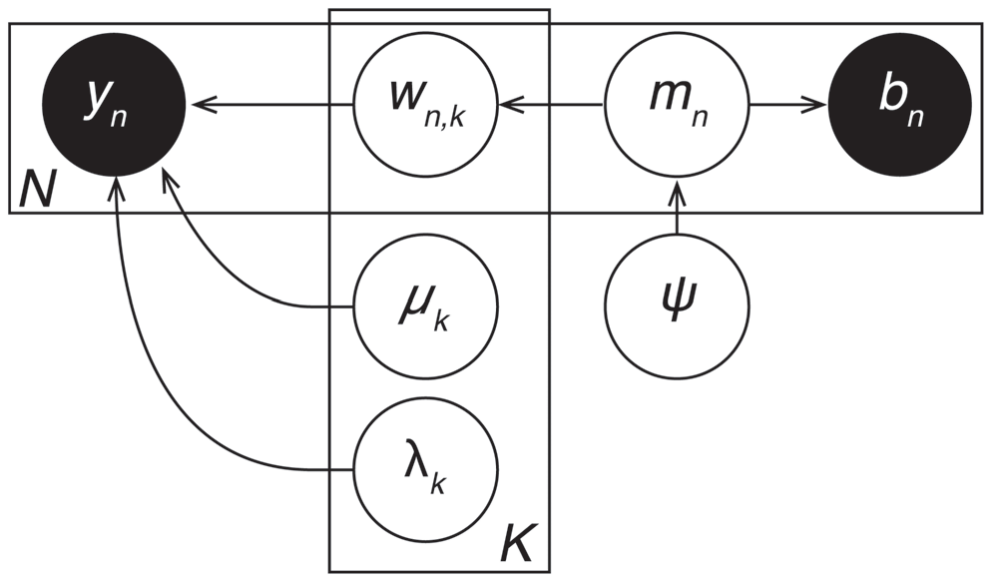
Integrating TFA into behavioral models. We show a proposed graphical model describing how TFA may be integrated into behavioral models. The model describes how a participant’s internal mental state during the 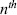 trial of an experiment (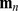) gives rise to the observed behavioral data (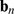) and the observed neural data (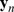). These mental states are drawn from a distribution controlled by a set of global variables, 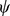. (The hyperparameters are omitted for notational compactness).

One way of integrating TFA into models of neural and behavioral data would be to vary the per-image source weights (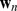) according to the internal cognitive states predicted by the behavioral model. As shown in Figure 9, the internal cognitive state 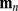 reflects what was happening in the participants’ mind during the 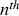 trial of the experiment, during which image 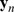 was collected and the participant exhibited behavior 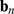. Each trial’s mental state is, in turn, drawn from a distribution controlled by a set of global variables, 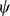, that define the general properties of the mental states participants are likely to exhibit. Combining neural and behavioral data into a common model allows these converging sources of information about participants’ internal mental states to jointly influence which brain structures are identified and which sequences of mental states are deemed most probable.

## Concluding Remarks

Topographic Factor Analysis (TFA) provides a means of automatically discovering a set of sources (and the interactions between those sources) that underlies an fMRI dataset. We presented an efficient algorithm that allowed us to apply TFA to fMRI datasets containing hundreds of images, each containing tens of thousands of voxels. In addition to yielding insights into complex datasets, we suggest that TFA may be incorporated into models that attempt to jointly account for neural and behavioral data.

## Supporting Information

Materials S1 **Supplemental equations and text.**
(PDF)

Movie S1 **A rotating view of the network displayed in** **Figure 1C** **.**
(AVI)

Movie S2 **A rotating view of the ANIMALS network displayed in** **Figure 7** **.**
(AVI)

Movie S3 **A rotating view of the INSECTS network displayed in** **Figure 7** **.**
(AVI)

Movie S4 **A rotating view of the TOOLS network displayed in** **Figure 7** **.**
(AVI)

Movie S5 **A rotating view of the VEHICLES network displayed in** **Figure 7** **.**
(AVI)
