# Supplementary material for: Topographic Factor Analysis: A Bayesian Model for Inferring Brain Networks from Neural Data
Source: PLoS One. 2014 May 7;9(5):e94914. doi: 10.1371/journal.pone.0094914 (PMC4012983; doi:10.1371/journal.pone.0094914)
Supplement: Materials S1 — Supplemental equations and text. (PDF) [file pone.0094914.s001.pdf]

Materials S1: Supplemental Materials for *Topographic Factor Analysis: a Bayesian model for inferring brain networks from neural data*

Jeremy R. Manning, Rajesh Ranganath, Kenneth A. Norman, and David M. Blei
Princeton University

1 Overview

This supplemental document provides additional details on equations referenced in the main text. These include $\log\, p(\mathbf{Y}, \mathbf{W}, \mathbf{M}, \mathbf{\Lambda})$, $\log\, q(\mathbf{W}, \mathbf{M}, \mathbf{\Lambda}|\alpha)$, and $\nabla_{\alpha_i} \log\, q(\mathbf{W}, \mathbf{M}, \mathbf{\Lambda}|\alpha)$. We also describe how we can sample from the variational distribution $q(\mathbf{W}, \mathbf{M}, \mathbf{\Lambda}|\alpha)$. Tables 1, 2, and 4 in the main text provide descriptions for each variable, hyperparameter, and variational parameter.

1.1 Computing $\log p(\mathbf{Y}, \mathbf{W}, \mathbf{M}, \mathbf{\Lambda})$

The log of the joint probability of the data and hidden variables is given by

$$\begin{aligned}\log p(\mathbf{Y}, \mathbf{W}, \mathbf{M}, \mathbf{\Lambda}) &= \log p(\mathbf{y}_{1\ldots N}, \mathbf{w}_{1\ldots N}, \mu_{1\ldots K}, \lambda_{1\ldots K})\\ &= \log p(\text{images}) + \log p(\text{weights}) + \log p(\text{centers}) + \log p(\text{widths})\\ &= \left[\sum_{n=1}^{N}\sum_{v=1}^{V} \log \mathcal{N}\left(y_{n,v}\middle|\mathbf{w}_n\mathbf{F}_v, \sigma_y^2\right)\right] + \left[\sum_{n=1}^{N}\sum_{k=1}^{K} \log \mathcal{N}\left(w_{n,k}\middle|\mu_w, \exp(\kappa_w)^{-1}\right)\right] +\\ &\quad \left[\sum_{k=1}^{K} \log \mathcal{N}\left(\mu_k\middle|\mathbf{c}, \exp(\kappa_\mu)^{-1}\mathbf{I}\right)\right] + \left[\sum_{k=1}^{K} \log \mathcal{N}\left(\lambda_k\middle|\mu_\lambda, \exp(\kappa_\lambda)^{-1}\right)\right]. \end{aligned} \tag{S1}$$

Here $\mathbf{F}_v$ refers to the vth column of the source image matrix $\mathbf{F}$. Note that when we compute the variational updates (Algorithm 2 in Table 5, main text), all of the hidden variables ($w_{1\ldots N,1\ldots K}$, $\mu_{1\ldots K}$, and $\lambda_{1\ldots K}$) are sampled from $q(\mathbf{W}, \mathbf{M}, \mathbf{\Lambda}|\alpha)$ as described below. We obtain one value of $\log p(\mathbf{Y}, \mathbf{W}, \mathbf{M}, \mathbf{\Lambda})$ for each set of samples we draw from $q(\mathbf{W}, \mathbf{M}, \mathbf{\Lambda}|\alpha)$.

1.2 Computing $\log q(\mathbf{W}, \mathbf{M}, \mathbf{\Lambda}|\alpha)$

The log of the variational distribution is given by

$$\begin{aligned}\log q(\mathbf{W}, \mathbf{M}, \mathbf{\Lambda}|\alpha) &= \log q(w_{1\ldots N,1\ldots K}, \mu_{1\ldots K,1\ldots D}, \lambda_{1\ldots K}|\alpha)\\ &= \log q(\text{weights}) + \log q(\text{centers}) + \log q(\text{widths})\\ &= \left[\sum_{n=1}^{N}\sum_{k=1}^{K} \log \mathcal{N}\left(w_{n,k}\middle|\tilde{\mu}_{w_{n,k}}, \exp(\tilde{\kappa}_{w_{n,k}})^{-1}\right)\right] +\\ &\quad \left[\sum_{k=1}^{K}\sum_{d=1}^{D} \log \mathcal{N}\left(\mu_{k,d}\middle|\tilde{\mu}_{\mu_{k,d}}, \exp(\tilde{\kappa}_{\mu_{k,d}})^{-1}\right)\right] + \left[\sum_{k=1}^{K} \log \mathcal{N}\left(\lambda_k\middle|\tilde{\mu}_{\lambda_k}, \exp(\tilde{\kappa}_{\lambda_k})^{-1}\right)\right] \end{aligned} \tag{S2}$$

Note that when we compute the variational updates (Algorithm 2 in Table 5, main text), all of the hidden variables ($w_{1...N,1...K}$, $\mu_{1...K}$, and $\lambda_{1...K}$) are sampled from $q(\mathbf{W}, \mathbf{M}, \mathbf{\Lambda}|\alpha)$ as described below. We obtain one value of $\log q(\mathbf{W}, \mathbf{M}, \mathbf{\Lambda}|\alpha)$ for each set of samples we draw from $q(\mathbf{W}, \mathbf{M}, \mathbf{\Lambda}|\alpha)$.

1.3 Computing $\nabla_{\alpha_i} \log q(\mathbf{W}, \mathbf{M}, \mathbf{\Lambda}|\alpha)$

Recall that the hidden variables include the per-image source weights $w_{1...N,1...K}$, the source centers $\mu_{1...K}$, and the source widths $\lambda_{1...K}$. Let α_i be a set of variational parameters that govern the (approximate) marginal posterior over one hidden variable in $\log\ p(\mathbf{Y}, \mathbf{W}, \mathbf{M}, \mathbf{\Lambda})$. For example, a given α_i might contain the mean $\tilde{\mu}_{w_{n,k}}$ and log precision $\tilde{\kappa}_{w_{n,k}}$ that govern the marginal posterior of $w_{n,k}$. To update α_i, we first need to sample each hidden variable from $q(\mathbf{W}, \mathbf{M}, \mathbf{\Lambda}|\alpha)$, and then we compute the gradient of $q(\mathbf{W}, \mathbf{M}, \mathbf{\Lambda}|\alpha)$ with respect to α_i, using those samples.

Because we constructed $q(\mathbf{W}, \mathbf{M}, \mathbf{\Lambda}|\alpha)$ to fully factorize, it is straightforward to compute the gradient of $q(\mathbf{W}, \mathbf{M}, \mathbf{\Lambda}|\alpha)$ with respect to a given α_i—we can simply compute the gradient with respect to α_i of the corresponding factor of $q(\mathbf{W}, \mathbf{M}, \mathbf{\Lambda}|\alpha)$, ignoring the other factors. Further, since the marginal distributions over the source weights, centers, and widths are all constructed to be univariate Gaussians (Equation S2), each of these gradients will have a similar form. Therefore, for notational convenience, we will find it useful to define a function for computing the derivative of a univariate log Gaussian, $\log \mathcal{N}\left(s \middle| \mu, \sigma^2\right)$, with respect to its mean parameter μ and its log precision parameter $\kappa = \log\left(\frac{1}{\sigma^2}\right)$:

$$z_1(s, \mu, \sigma^2) = \frac{\partial}{\partial \mu} log\ \mathcal{N}\left(s \middle| \mu, \sigma^2\right) = \frac{s - \mu}{\sigma^2} \tag{S3}$$

$$z_2(s, \mu, \sigma^2) = \frac{\partial}{\partial \kappa} log\ \mathcal{N}\left(s \middle| \mu, \sigma^2\right) = \frac{1}{2} - \frac{1}{2}(s - \mu)^2 \exp(\kappa). \tag{S4}$$

These functions allow us to easily define the gradients of $q(\mathbf{W}, \mathbf{M}, \mathbf{\Lambda}|\alpha)$ with respect to each α_i. To keep track of each set of variational parameters, we will label the sets according to the hidden variable whose marginal posterior they govern:

1.4 $w_{n,k}$

$$\nabla_{\tilde{\mu}_{w_{n,k}}} \log q(\mathbf{W}, \mathbf{M}, \mathbf{\Lambda}|\alpha) = z_1\left(w_{n,k}, \tilde{\mu}_{w_{n,k}}, \exp(\tilde{\kappa}_{w_{n,k}})^{-1}\right) \tag{S5}$$

$$\nabla_{\tilde{\kappa}_{w_{n,k}}} \log q(\mathbf{W}, \mathbf{M}, \mathbf{\Lambda}|\alpha) = z_2\left(w_{n,k}, \tilde{\mu}_{w_{n,k}}, \exp(\tilde{\kappa}_{w_{n,k}})^{-1}\right) \tag{S6}$$

1.5 $\mu_{k,d}$

$$\nabla_{\tilde{\mu}_{\mu_{k,d}}} \log q(\mathbf{W}, \mathbf{M}, \mathbf{\Lambda}|\alpha) = z_1\left(\mu_{k,d}, \tilde{\mu}_{\mu_{k,d}}, \exp(\tilde{\kappa}_{\mu_{k,d}})^{-1}\right) \tag{S7}$$

$$\nabla_{\tilde{\kappa}_{\mu_{k,d}}} \log q(\mathbf{W}, \mathbf{M}, \mathbf{\Lambda}|\alpha) = z_2\left(\mu_{k,d}, \tilde{\mu}_{\mu_{k,d}}, \exp(\tilde{\kappa}_{\mu_{k,d}})^{-1}\right) \tag{S8}$$

1.6 λ_k

$$\nabla_{\tilde{\mu}_{\lambda_k}} \log q(\mathbf{W}, \mathbf{M}, \mathbf{\Lambda}|\alpha) = z_1\left(\lambda_k, \tilde{\mu}_{\lambda_k}, \exp(\tilde{\kappa}_{\lambda_k})^{-1}\right) \tag{S9}$$

$$\nabla_{\tilde{\kappa}_{\lambda_k}} \log q(\mathbf{W}, \mathbf{M}, \mathbf{\Lambda}|\alpha) = z_2\left(\lambda_k, \tilde{\mu}_{\lambda_k}, \exp(\tilde{\kappa}_{\lambda_k})^{-1}\right) \tag{S10}$$

1.7 Sampling from $q(\mathbf{W}, \mathbf{M}, \mathbf{\Lambda}|\alpha)$

Sampling from $q(\mathbf{W}, \mathbf{M}, \mathbf{\Lambda}|\alpha)$ yields one value for each of the hidden variables, including the per-image source weights $w_{1\ldots N,1\ldots K}$, the source centers $\mu_{1\ldots K}$, and the source widths $\lambda_{1\ldots K}$. Because $q(\mathbf{W}, \mathbf{M}, \mathbf{\Lambda}|\alpha)$ fully factorizes, each of these variables are drawn independently, as shown in Algorithm S1. To draw M samples from $q(\mathbf{W}, \mathbf{M}, \mathbf{\Lambda}|\alpha)$, we simply repeat this procedure M times.

Algorithm S1: Sampling from $q(\mathbf{W}, \mathbf{M}, \mathbf{\Lambda}|\alpha)$.

for $k = 1$ **to** K **do**
- **for** $d = 1$ **to** D **do**
  - Pick dimension d of source k's center $\mu_{k,d} \sim \mathcal{N}\left(\tilde{\mu}_{\mu_{k,d}}, \exp(\tilde{\kappa}_{\mu_{k,d}})^{-1}\right)$
- **end**
- Pick source k's width $\lambda_k \sim \mathcal{N}\left(\tilde{\mu}_{\lambda_k}, \exp(\tilde{\kappa}_{\lambda_k})^{-1}\right)$
- **for** $n = 1$ **to** N **do**
  - Pick source k's weight in image n $w_{n,k} \sim \mathcal{N}\left(\tilde{\mu}_{w_{n,k}}, \exp(\tilde{\kappa}_{w_{n,k}})^{-1}\right)$
- **end**

end
